# Enhancing developmental and reproductive toxicity knowledge: A new AOP stemming from glutathione depletion

**DOI:** 10.1016/j.crtox.2023.100124

**Published:** 2023-09-15

**Authors:** Alun Myden, Susanne A. Stalford, Adrian Fowkes, Emma White, Akihiko Hirose, Takashi Yamada

**Affiliations:** a Lhasa Limited, Granary Wharf House, 2 Canal Wharf, Leeds LS11 5PS, United Kingdom; b Division of Risk Assessment, Center for Biological Safety and Research, National Institute of Health Sciences, 3-25-26 Tonomachi, Kawasaki-ku, Kawasaki 210-9501, Japan

**Keywords:** Adverse outcome pathway, Glutathione, Male fertility toxicity, Structure activity relationship

## Abstract

Integrated approaches to testing and assessments (IATAs) have been proposed as a method to organise new approach methodologies in order to replace traditional animal testing for chemical safety assessments. To capture the mechanistic aspects of toxicity assessments, IATAs can be framed around the adverse outcome pathway (AOP) concept. To utilise AOPs fully in this context, a sufficient number of pathways need to be present to develop fit for purpose IATAs. *In silico* approaches can support IATA through the provision of predictive models and also through data integration to derive conclusions using a weight-of-evidence approach. To examine the maturity of a developmental and reproductive toxicity (DART) AOP network derived from the literature, an assessment of its coverage was performed against a novel toxicity dataset. A dataset of diverse compounds, with data from studies performed according to OECD test guidelines TG-421 and TG-422, was curated to test the performance of an *in silico* model based on the AOP network – allowing for the identification of knowledge gaps within the network. One such gap in the knowledge was filled through the development of an AOP stemming from the molecular initiating event ‘glutathione reaction with an electrophile’ leading to male fertility toxicity. The creation of the AOP provided the mechanistic rationale for the curation of pre-existing structural alerts to relevant key events. Integrating this new knowledge and associated alerts into the DART AOP network will improve its coverage of DART-relevant chemical space. In addition, broadening the coverage of AOPs for a particular regulatory endpoint may facilitate the development of, and confidence in, robust IATAs.

## Highlights

• The performance of a DART AOP model was assessed using an *in vivo* dataset.
• Mechanistic gaps within the model were investigated.
• An AOP for glutathione depletion leading to male fertility toxicity was developed.
• Structural alerts were associated to the AOP, broadening the DART AOP model.

## Graphical abstract

**Figure:**
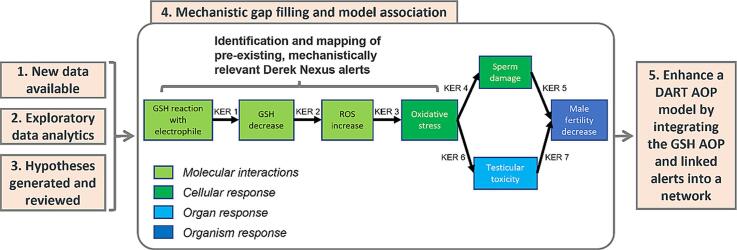

## 1 Introduction

With the global drive to reduce animal testing for safety assessments, the need for alternative new approach methodologies (NAMs) (encompassing *in vitro* and *in silico* methods) is of paramount importance. To this end, progress has been made within the cosmetics industry for skin sensitisation testing – motivated by the 2013 European Union ban on testing of new cosmetic products on animals (European Commission, 2013). This move has led to the establishment of defined approaches, utilising assays and models which measure events relevant to the established mode-of-action for skin sensitisation (Kleinstreuer et al., 2018, Macmillan and Chilton, 2019). For endpoints where no suitable defined approach exists, integrated approaches to testing and assessment (IATA) are being investigated as flexible methods of combining alternatives to animal testing, in order to confidently replace the traditional models (Casati, 2018). However, progress towards the transition from *in vivo* studies to alternative tests for the regulatory assessment of other endpoints are less advanced, which in part may be due to greater numbers of mechanisms relevant to other toxicity endpoints. Regardless of the endpoint, it appears that multiple NAMs are required to generate sufficient data to satisfy the risk assessment without the need to rely on traditional animal tests (Krewski et al., 2010).

For developmental and reproductive toxicity (DART), *in vivo* studies for assessment of various endpoints are well established (EMA/CHMP/ICH/544278/1998, 2020). In recent years, many NAMs have been described in the literature (e.g. embryonic stem cell assay, rat limb bud micromass assay, rat whole-embryo culture assay, zebrafish assay and a stem cell based biomarker assay), and validation studies of some of these assays have been undertaken (Genschow et al., 2004, Genschow et al., 2002, Jamalpoor et al., 2022, Panzica-Kelly et al., 2012, Paquette et al., 2008, Piersma et al., 2004, Spielmann et al., 2004). However, no suitable battery of *in vitro* and/or *in silico* methods, nor IATA, have been approved for regulatory use to replace the animal models (Clements et al., 2020). One potential barrier to regulatory approval is that NAMs can generate a lot of complex data (especially when used in combination as part of an IATA), and the mechanistic understanding of these assays may be difficult to contextualise. Therefore, a framework to organise and contextualise the data for hazard or risk assessments needs to be established to aid in the decision-making process – this could be achieved using adverse outcome pathways (AOPs) (Clements et al., 2020).

**Fig. 1:**
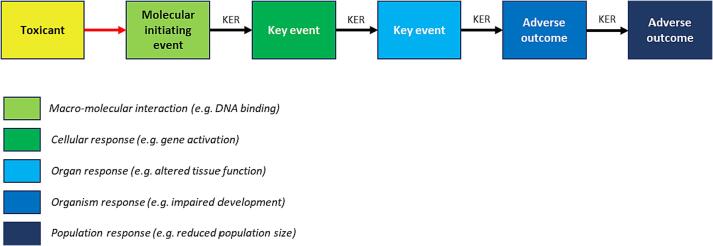
The adverse outcome pathway concept. Figure adapted from Ankley et al, 2010 (Knapen et al., 2015). KER, key event relationship.

The AOP concept is a formalised approach to documenting toxicity pathways (Ankley et al., 2010). AOPs are defined by key events (KEs), starting with the molecular initiating event (MIE) such as a compound interacting with a biological entity (e.g. an enzyme or receptor). This often triggers a chain of KEs, linked by key event relationships, until an adverse outcome (AO) is reached (Fig. 1). KEs often proceed up the levels of biological organisation through cellular, tissue and organ-based events – ultimately reaching an organism or population-based AO. Assays or predictions can be linked to KEs within the pathway, to act as entry points to the AOP and indicating relevant regulatory endpoints and mechanisms of interest to safety assessors.

Using AOPs as part of an IATA strategy has been advocated for by the Organisation for Economic Co-operation and Development (OECD) (OECD, 2017). Networks of AOPs incorporating known mechanisms leading to an adverse outcome may be required in order to successfully utilise AOPs in IATAs. Progress to this end has been made for specific endpoints such as thyroid hormone disruption (within the ecotoxicity domain) (Knapen et al., 2020), and a recently published network of (mammalian-relevant) AOPs for carcinogenicity (Cayley et al., 2022). *In silico* models can form part of an IATA (OECD, 2020), and therefore mapping relevant *in silico* models to AOPs could increase their use in an IATA and also introduce a predictive entry point to potentially relevant AOPs. Derek Nexus is a commercially available *in silico* tool, which comprises structure activity relationship (SAR)-based alerts developed for numerous toxicity endpoints (Marchant et al., 2008). Currently, a network of mammalian-relevant AOPs for DART endpoints is being developed (e.g. CYP19 related pathways (Myden et al., 2022)). A model based on mechanistically relevant structural alerts mapped to the AOP network can be used to make predictions for novel compounds – this is referred to as the ‘AOP model’ throughout this publication. This model allows for the prediction of DART and is able to gauge the coverage of the AOP network.

High-quality data is essential to support the validation of predictive models, and a dataset containing many unique compounds can enhance the validation process by assessing the model’s performance over wider areas of chemical space. Such exercises can help to find predictive gaps that other datasets may not have identified. Attention could then be focused on these gaps in order to improve the predictive model. For a model based on AOPs, the identified gaps could be filled by first developing new AOP knowledge and then using the mechanistic understanding within the pathway to associate additional relevant predictions. In order to obtain and utilise high-quality datasets, a collaborative project was initiated between the National Institute of Health Sciences (NIHS) and Lhasa Limited to investigate the coverage of an AOP network for chemicals used in the industrial chemical sector. NIHS data from studies conducted in accordance with OECD test guidelines TG-421 (‘Reproduction/Developmental Toxicity Screening Test’) and TG-422 (‘Combined Repeated Dose Toxicity Study with the Reproduction/Developmental Toxicity Screening Test’) were donated to Lhasa in 2018 for analysis to identify gaps in knowledge and support new AOPs – this dataset has subsequently been published (Yamada et al., 2021). Based on the false negatives within the dataset (i.e. active compounds in the dataset which the AOP model failed to identify), a review of the mechanistic gaps within the AOP network was undertaken, and coverage was improved through the integration of additional AOPs. In this manuscript, an AOP for glutathione depletion leading to impairment of male fertility is presented, along with the mapping of relevant *in silico* models. Together, this resulted in new knowledge and the demonstration of how this can be used to enhance future predictive systems.

## 2 Methods

### 2.1 Data and analysis

A dataset containing 397 study results for compounds tested according to OECD test guidelines TG-421 and TG-422 (hereafter referred to as TG-421 and TG-422 studies) (OECD, 2016, OECD, 2015, Yamada et al., 2021) was developed by the NIHS and shared with Lhasa. The records within the dataset were curated and associated structural information was standardised using in-house transformation rules to enable data mining (Fourches et al., 2010). The dataset was grouped by unique structures which resulted in a dataset of 394 unique structures.

Study observations in the data were recorded at a granular level of specific observations (e.g. pre-implantation loss increase and abnormal oestrous cycle). These observations were grouped based on whether the toxicity was observed in the dams or the offspring (Yamada et al., 2021). To support the identification of activity relationships, a series of rules were implemented to classify compounds with respect to toxicity. TG-421 and TG-422 state that the highest tested dose (HTD) should induce toxicity (OECD, 2016, OECD, 2015). With this in mind, the assumption was made that toxicity at the HTD may not be DART related – compounds which caused DART selectively, rather than secondary to another event, could be termed ‘selective toxicants’, whereas compounds which gave rise to DART in the presence of systemic toxicity were termed ‘non-selective toxicants’. Therefore, based on the lowest observed-effect level (LOEL) and highest tested dose (HTD) provided, the compounds were categorised as follows for each of the grouped endpoints:

• Endpoint LOEL < HTD => ‘Selective toxicant’
• Endpoint LOEL = HTD => ‘Non-selective toxicant’
• Endpoint LOEL not observed => ‘Non-toxicant’

Table 1 represents the structure of the model-ready dataset after curation, and shows the distribution of compound classes for the two high-level groupings (dams and offspring) (Yamada et al., 2021).

**Table 1:** Number of toxicants per dam or offspring observation grouping in the curated dataset.

Observation grouping	Total	Selective toxicants	Non-selective toxicants	Non-toxicants
Dams	394	39	102	253
Offspring	394	33	117	244

### 2.2 Validation of *in silico* models

**Fig. 2:**
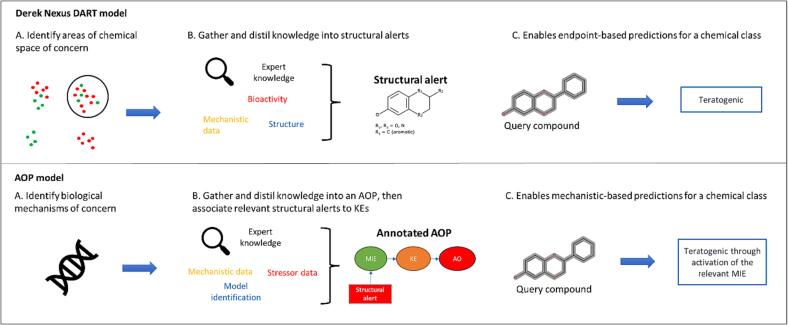
Representation of the Derek Nexus DART model and the AOP model. Sections A and B depict the general process for how each model was constructed, and Section C reflects the outputs of each model. For the Derek Nexus model, the query compound has matched a structural alert for the endpoint of teratogenicity. For the AOP model, the query compound has matched a structural alert associated to an AOP. AOP, adverse outcome pathway; DART, developmental and reproductive toxicity; KEs, key events; MIE, molecular initiating event.

The curated dataset (Table 1) was used to validate two DART prediction models (Fig. 2). One model, the ‘Derek Nexus DART model’, used Derek Nexus (Marchant et al., 2008) alerts for the DART endpoints of developmental toxicity, teratogenicity, and testicular toxicity. These alerts are primarily trained on data derived from toxicity studies. The second model was the ‘AOP model’, which used a broader set of alerts from Derek Nexus. This included Derek Nexus alerts that highlighted modes of action (in the alert comments) which were relevant to an AOP network under construction within Lhasa Limited.

The Derek Nexus DART model and the AOP model were both validated using the dams- and offspring-related observations in the donated dataset (Table 1). For each case, the relevant models were selected. For example, the teratogenicity and developmental toxicity models were used when validating against the offspring endpoint. Two scenarios were used to generate performance metrics for the models against the donated dataset. The first scenario resulted in non-selective toxicants being classified as toxicants (‘Included’) and the second scenario involved exclusion of non-selective toxicants from the test dataset (‘Excluded’). In each instance the following parameters (based on Cooper statistics (Cooper et al., 1979)) were calculated:

• Sensitivity = true positives/(true positives + false negatives)
• Specificity = true negatives/(true negatives + false positives)
• Balanced accuracy = (sensitivity + specificity)/2
• Positive predictivity = true positives/(true positives + false positives)
• Negative predictivity = true negatives/(true negatives + false negatives)

### 2.3 Analysis of knowledge gaps

Further analysis of the performance of the AOP model focused on identifying areas of toxicological concern that were not adequately covered by the model. The aim of this analysis was to identify patterns within the toxicants in the donated dataset which were incorrectly predicted by the AOP model (e.g. false negatives) for either the dam- or offspring-related observations. In order to achieve this, patterns in mechanistic similarity or structural similarity were investigated. Examples of the methods used to identify patterns in the false negative compounds included:

• Emerging pattern mining (Sherhod et al., 2014)
  o Algorithms compare the prediction results to experimental values for each compound in the dataset and then identify structural features of toxicants not covered by the model.
• Derek Nexus
  o Structural alerts predicting for other endpoints were used to profile compounds in the donated dataset. The results were analysed to identify putative toxicophores and mechanisms associated with DART.
• QSAR models
  o Biofingerprints for selective toxicants were generated using statistical models provided by ChEMBL (Mendez et al., 2019). These predictions may highlight protein targets which could be mediating the adverse outcome. Targets already described in the AOP model were excluded and the remaining targets were reviewed for the identification of any associated DART liabilities.

Expert review of the false negatives within the NIHS dataset (based on the outputs of the AOP model) were then investigated to identify patterns which could be followed up for AOP development. During expert review, additional toxicity data for structurally similar compounds was reviewed and putative mechanisms of toxicity were documented.

### 2.4 AOP development

Prioritised signals from the data mining were then examined to support the construction of a DART-relevant AOP. The approach taken to build a qualitative AOP focused on describing the main evidence that indicated a causal relationship between a biological target (related to these compounds) and an adverse outcome. The AOP was synthesised using a literature-based approach where searches focused on identifying studies that provided empirical evidence and biological plausibility for:

• The physiological role of the target, including its function in supporting normal development or fertility
• Toxicity of marketed pharmaceuticals and chemicals associated with the target
• Identification of relevant mechanistic data
• Potential uncertainties in the pathway
• Domain of applicability, including sex, species, and life stage

## 3 Results

### 3.1 Performance of *in silico* models

**Fig. 3:**
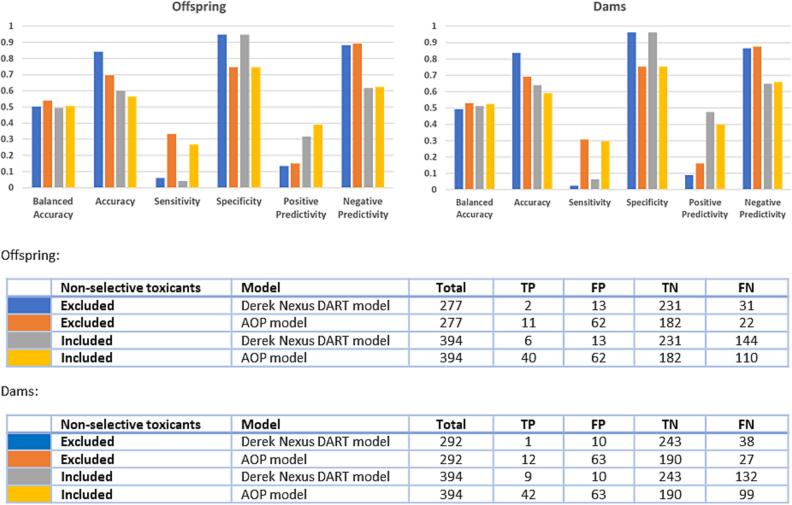
Performance of the *in silico* models against the dam and offspring grouped activity calls from the donated data. The colours in the bar charts represent the data and model combinations indicated in the tables. TP, true positive; FP, false positive; TN, true negative; FN, false negative.

The curated toxicity dataset (Table 1) was screened against two models: a Derek Nexus DART model, and the AOP model (Fig. 3). The validation results showed similar trends in model performance for both the dams- and offspring-related observations, and whether non-selective toxicants were included or not. An increase in sensitivity was observed for the AOP model compared to the Derek Nexus DART model. This increase was associated with a similar decrease in specificity for the AOP model, resulting in comparable balanced accuracies between the two models (approximately 50%). The increase in sensitivity of the AOP model is driven by the additional mechanisms described in this model, allowing for more structural alerts to be leveraged. Although large improvements were observed in the sensitivity of the AOP model, compared to the Derek Nexus DART model, false negatives were still present. This indicates either predictive gaps in the alerts associated to the model or a mechanistic knowledge gap – where a suitable AOP had not yet been captured within the developing AOP network.

### 3.2 Analysis of knowledge gaps

To identify areas where new knowledge could improve the model, clusters of false negatives in the donated dataset with respect to the AOP model were mined. These were examined to identify areas of chemical/biological space not presently described in the model. One such cluster of interest related to a set of 12 compounds which were identified as potential gonadotropin-releasing hormone receptor (GnRHR) binders. Six of these compounds were toxicants and six were non-toxicants in the donated dataset. These compounds were predominantly nitroaromatics, and were identified using a publicly available QSAR model (ChEMBL, 2018, ChEMBL, 2014, Mendez et al., 2019). Due to the literature surrounding the importance of the GnRHR in regulating fertility (Millar et al., 2004), these 12 compounds were taken forward for further analysis to assess whether the compounds could support the development of an AOP.

### 3.3 Cluster of nitroaromatic compounds

The cluster of 12 (predominantly nitroaromatic) compounds which were identified as being potentially relevant to GnRHR binding, and are currently not captured within the AOP model, are described in Table 2. Six of these compounds were classified as toxicants (five of which were non-specific), whilst six were classified as non-toxicants. Four of the non-toxicants had relatively low highest tested doses of < 100 mg/kg/day – this may indicate that other signs of toxicity/systemic toxicity were observed at these doses.

**Table 2:** A cluster of 12 compounds not captured by the AOP model which were identified by an external QSAR model as being potentially relevant to GnRHR binding.

Name and CAS number	Structure	Toxicity call from dataset	Highest tested dose (HTD) and/or Lowest observed effect level (LOEL)
2-methyl-5-nitrobenzenesulfonic acid (Pntos) 121-03-9		Non-specific offspring toxicant	LOEL/HTD – 700 mg/kg/day
3-methyl-4-nitrophenol (PNMC) 2581-34-2		Non-toxicant	HTD – 30 mg/kg/day
2,4-dinitrophenol (DNP) 51-28-5		Non-specific offspring toxicant	LOEL/HTD – 30 mg/kg/day
1,3-Dichloro-4-nitrobenzene (2,4-DCNB) 611-06-3		Non-specific offspring and dam toxicant	LOEL/HTD – 200 mg/kg/day
4-nitrophenol (4-NP) 824-78-2		Non-toxicant	HTD – 400 mg/kg/day
9H-carbazole 86-74-8		Dam toxicant and non-specific offspring toxicant	Dam LOEL – 100 mg/kg/day Offspring LOEL/HTD – 400 mg/kg/day
2,4,6-trinitrophenol (picric acid) 88-89-1		Non-toxicant	HTD – 45 mg/kg/day
1,4-dichloro-2-nitrobenzene (2,5-DCNB) 89-61-2		Non-specific offspring and dam toxicant	LOEL/HTD – 200 mg/kg/day
1-chloro-2,4-dinitrobenzene (CDNB) 97-00-7		Non-toxicant	HTD – 30 mg/kg
4-*tert*-butyltoluene (PTBT) 98-51-1		Offspring and dam toxicant	Dams LOEL – 15 mg/kg/day Offspring LOEL – 5 mg/kg/day
4-*tert*-butylphenol (PTBP) 98-54-4		Non-toxicant	HTD – 200 mg/kg/day
1,2-dichloro-4-nitrobenzene (DCNB) 99-54-7		Non-toxicant	HTD – 100 mg/kg/day

As many of these compounds are reprotoxicants (selective and non-selective) and are chemically similar to compounds indicated as potential GnRHR binders, a literature search was conducted to achieve two goals. The first was to evaluate support for the predicted biological activity, and the second was to determine whether there was sufficient evidence available within the public domain to support the development of a suitable AOP. Based on this investigation, no evidence linking the 12 compounds to GnRHR binding could be identified within the public domain. This finding was not unexpected, as the structures in Table 2 are much smaller than the natural substrate of the GnRHR – a ten-amino-acid peptide (Marques et al., 2000). Our assumption is that the publicly available QSAR model (ChEMBL, 2018, ChEMBL, 2014, Mendez et al., 2019) classified these chemicals as binders due to the presence of the aromatic structures (Table 2) within a number of GnRHR binders in the ChEMBL database. Instead, as reported in the following sections, the most abundant mechanistic data related to DART appeared to be linked to glutathione depletion and oxidative stress-related toxicity (Kovacic and Jacintho, 2001, Kovacic and Somanathan, 2014). This mechanism applied to the nitroaromatic compounds within the cluster of 12, and the majority of the supporting mechanistic information related to testicular toxicity as opposed to female fertility and teratogenicity (relevant studies are outlined in the following sections). Therefore, to reduce the data/knowledge gap within our DART AOP network, we opted to develop a putative AOP for electrophilic reactions with glutathione leading to male fertility toxicity – supported by data in the public domain for nitroaromatic compounds. The decision to focus our efforts on developing an AOP for the male fertility DART endpoint was based on two key factors: 1) documenting the most confident DART AOP based on the cluster of nitroaromatic compounds would result in the highest confidence in their subsequent uses (e.g. making part of an enhanced predictive AOP model or framing the development of an IATA); 2) once completed, the AOP may provide insights and a starting point for the development of pathways for female fertility toxicity and developmental toxicity endpoints.

### 3.4 Nitroaromatics and male fertility toxicity

This section focuses on *in vivo* male fertility studies for nitroaromatic compounds. Several nitroaromatic compounds have been shown to cause testicular toxicity and sperm damage when tested in animals (details of such studies are described below and summarised in Table 3). As a class, nitroaromatic compounds have been proposed to cause toxicity through oxidative stress (Kovacic and Jacintho, 2001, Kovacic and Somanathan, 2014). As oxidative stress is a mechanism which can occur in many tissue types, it is associated with multiple toxicity endpoints – such as liver and kidney toxicity (Yamazaki et al., 2005b). Many of the compounds described below have been used to manufacture a diverse range of products such as plastics, dyes and pesticides (Koizumi et al., 2001, Matsumoto et al., 2008, Zhang et al., 2016) and, therefore, are produced in high volumes (e.g. are listed on the OECD High Production Volume Chemical Table (which means > 1,000 tonnes of the chemical in question is manufactured per year in at least one OECD country)) (Koizumi et al., 2001). Additionally, nitroaromatic compounds can be found in exhaust fumes (Li et al., 2006). Understanding the toxic implications of these compounds is very important due to the abundance of, and potential exposure to, these chemicals in everyday life.

**Table 3:** Summary of the available evidence linking nitroaromatic compounds to glutathione depletion, oxidative stress and testicular toxicity. Key: + = studies support the observation, – = studies do not support the observation, blank = no relevant studies were identified. Compounds marked with a superscript ‘a’ were also in the NIHS donated dataset.

	GSH decrease	ROS increase	Oxidative stress	Sperm damage	Testicular Toxicity
Dinoseb^a^				+	+
2,5-DCNB^a^	+			+	+
DNAN					+
DNOC^a^				+	–
DNP				–	–
TNT^a^				+	+
NB^a^				+	+
1,3-DNB^a^	+			+	+
2,6-DNT^a^				+	+
2,4-DNT^a^				+	+
Picric acid^a^				+	+
PNMC	+	+	+	+	+
4-NP^a^	+	+	+	+	+

#### 2-*sec*-Butyl-4,6-dinitrophenol (Dinoseb)

A review of studies in which dinoseb was dosed in rodents concluded that dinoseb is a testicular toxicant (Matsumoto et al., 2008). The following results were used to support this conclusion (Matsumoto et al., 2008):

• Reduced and abnormal sperm motility observed in rats when given daily by gavage.
• Reduced sperm counts, reduced morphologically normal sperm, adjusted weight of seminal vesicle, testes and epididymis, and reduced sperm content of caudae and vasa deferentia in several rat feeding studies. One feeding study also resulted in reduced fertility.
• Testicular atrophy or degeneration with hypospermatogenesis were observed in a two-year mouse feeding study.

Whilst the majority of the studies reviewed demonstrated dinoseb to be a testicular toxicant, the authors noted that a three-generation rat study showed no effects on fertility (Matsumoto et al., 2008).

#### 4,6-Dinitro-o-cresol (DNOC)

A review article concluded that DNOC is a less potent testicular toxicant than dinoseb (Matsumoto et al., 2008). Evidence highlighting the toxicity of DNOC includes (Matsumoto et al., 2008):

• Gavage dosing of DNOC on five consecutive days in rats led to reduced sperm motility, increased peeled and tailless sperm, but did not have an effect on histopathology of the testes.
• Consecutive dosing by gavage or intraperitoneal injection in mice had no effect on sperm morphology 30 days after treatment – indicating no long-term effect.
• A 90-day feeding study in rats resulted in reduced spermatogenesis and reduced relative organ weights of testes and prostate. However, a six-month feeding study found no effects on the testes.
• A two-generational study reported no effects on fertility.
• A single dose intraperitoneal injection in male mice resulted in germ cell damage. These mice were mated with untreated females for eight weeks and, after six weeks, there was a decrease in the number of living embryos.

#### 2,4-Dinitrophenol (DNP)

A review article concluded that DNP is not a testicular toxicant, which may be due to its rapid clearance (Matsumoto et al., 2008). Consecutive daily doses of DNP in rats did not result in testicular toxicity, with no effects on sperm or sex organs observed in multiple studies. Six-month feeding studies in rats and dogs failed to detect any testicular toxicity. One feeding study in rats reported testicular atrophy and small testes; however, the authors suggested that these may be secondary effects rather than observations of toxicity (Takahashi et al., 2009). Additionally, it was found that treating rats with DNP resulted in significant reduction in spermatogonia during spermatogenesis, compared to controls (Takahashi et al., 2009).

#### Trinitrotoluene (TNT)

A 13-week dietary study, examining the effects of TNT in rats, demonstrated that TNT caused: testicular atrophy at the highest dose level, degeneration of the seminiferous tubular epithelium, hyperplasia of interstitial Leydig cells, and diffuse intertubular oedema (Levine et al., 1984). At lower doses, less severe lesions were seen and included a decrease in (or absence of) spermatozoa, spermatids and spermatocytes. The liver and blood are prone to TNT-induced toxicity, with reduced body weight gain and anaemia also being observed – as well as other signs of systemic toxicity.

#### Nitrobenzene (NB)

A rat study with single doses of NB concluded that the liver and testes are the main target organs of toxicity for the compound (Bond et al., 1981). At higher doses, lesions of the seminiferous tubules were observed, along with destruction of the spermatocytes and decreased number of spermatozoa in the epididymis. In the liver, enlarged nucleoli and centrilobular necrosis were observed. There was a dose-dependent increase in methaemoglobin levels in rats following NB administration, but the cause of liver and testicular damage was believed to be due to a direct effect of the compound on the organs.

#### 1,3-Dinitrobenzene (1,3-DNB)

Rats were exposed to 1,3-DNB in drinking water for eight weeks (Cody et al., 1981). Mortality, reduced body weight and enlarged spleens were observed. Testicular atrophy was also observed, as well as decreased spermatogenesis and collapsed seminiferous tubules. A reduction in the haemoglobin content of blood was seen in a 16-week exposure study, as well as decreased weight of testes. 1,3-DNB has also been shown to decrease mitochondrial glutathione concentrations (Creasy and Chapin, 2018).

#### 2,6-Dinitrotoluene (2,6-DNT)

Studies spanning 13 weeks in dogs and rats resulted in testicular atrophy and reduced spermatogenesis (Rickert et al., 1984). In mice dosed for four weeks, reduced spermatogenesis was also observed.

#### 2,4-Dinitrotoluene (2,4-DNT)

Rats exposed to 2,4-DNT for 13 weeks showed severe testicular atrophy and impaired spermatogenesis. Dogs exposed for 13 weeks also showed a decrease in spermatogenesis (Rickert et al., 1984). A mouse study did not result in any testicular lesions. In a two-year study with 2,4-DNT, dogs did not present any testicular lesions, while in rats and mice, spermatogenesis was impaired and testicular atrophy was seen. A three-generation study in rats, where both males and females of each generation were exposed to 2,4-DNT in the diet, found no effects on reproduction (Rickert et al., 1984).

#### 3-Methyl-4-nitrophenol (PNMC)

Mice exposed intraperitoneally to PNMC, at a single dose of 100 mg/kg, demonstrated significant damage to the seminiferous tubules (Bu et al., 2012). In addition, a 40% loss of round germ cells, no detectable long spermatozoa and testicular atrophy was also observed. These findings were also coupled with a decrease in glutathione concentrations. Co-administration of PNMC and an antioxidant (quercetin) alleviated the toxicity and the decrease in glutathione.

#### 4-Nitrophenol (4-NP)

Rats dosed with 4-nitrophenol demonstrated a significant decrease in testicular glutathione (GSH), as well as a significant increase in testicular hydrogen peroxide (Zhang et al., 2015). This increase in hydrogen peroxide was alleviated by co-administration with an antioxidant. In rats examined 7 days after treatment with a single dose, intratesticular injection of 4-nitrophenol resulted in testicular toxicity such as damage to the germinal epithelium, as well as abnormal sperm development (Zhang et al., 2016). In this study, oxidative stress was observed via an increase in activity of superoxide dismutase (SOD), catalase (CAT) and glutathione peroxidase (GSH-Px), as well as an increase in H_2_O_2_ and a decrease in GSH concentrations. Co-administration of 4-NP with the antioxidant phytosterin resulted in less significant changes in some of the above proteins and chemicals. The Nrf2 pathway was identified as the mechanism of 4-NP-mediated oxidative stress. As with other compounds described in this section, 4-nitrophenol is a non-selective toxicant known to affect other organs such as the liver and kidney.

#### 2,4,6-Trinitrophenol (picric acid)

In a comparative study in which newborn and 5-week-old rats were dosed orally with picric acid, male fertility toxicity was identified in the 5-week-old group (Takahashi et al., 2004). The 5-week-old rats were given picric acid in doses of 0, 4, 20 and 100 mg/kg per day for two weeks. Testicular toxicity as well as haemolytic anaemia were observed in the 100 mg/kg per day dose group. Testicular toxicity manifested as small testes with diffuse atrophy and a 21% decrease in epididymis weight, relative to the control group. These signs of testicular toxicity persisted even after a recovery period. Decreased sperm and lumen were also observed in the effected epididymis. Interestingly, similar signs of testicular toxicity were not observed in the newborn rats treated with picric acid. The authors suggest that the toxicity may result from oxidation-based mechanisms which may not be present in the newborn rats (which have a less mature metabolism).

#### 1,4-Dichloro-2-nitrobenzene (2,5-DCNB)

A study in which rats and rabbits were dosed daily for 2 weeks with 2,5-DCNB demonstrated the compound to be a testicular toxicant (Yamazaki et al., 2005b). Both species were treated orally (via animal feed containing 2,5-DCNB) in concentration groups of 1250, 2500, 5000 and 10000 ppm per day. Testicular weights were significantly decreased in rats within the 5000 and 10000 ppm dose groups and in mice within the 10000 ppm dose group. As with other nitroaromatic compounds described in this manuscript, other signs of toxicity were also observed, such as liver toxicity. Of the organs examined, the kidneys appear to be the most sensitive organ to histopathological changes, however testicular lesions were observed in male rats treated at 5000 ppm. At this concentration, a decrease in germ cells was also observed (decreased spermatocytes and a decreased sperm count in the epididymis). In mice, germ cell necrosis was observed in the 5000 ppm dose group.

Within the same manuscript, a study (reported in Japanese) and undertaken by a different group is also described. In this study, similar results were observed in a 28-day repeat dose study in rats – testicular toxicity, as observed as degeneration of the seminiferous epithelium in the testicles, was observed at a dose of 60 mg/kg per day.

A long term (13-week) oral toxicity study with 2,5-DCNB was also conducted in rats and mice and similar toxicities were observed (Yamazaki et al., 2005a). In this study, the maximum dose of 2,5-DCNB was 7500 ppm and germ cell necrosis was observed in rat testes from 2222 ppm. Lack of sperm in the epididymides was observed at 3333 ppm. In mice, these observations were made at 7500 ppm.

A study of the urine of rats treated with 2,5-DCNB identified *N*-acetyl-*S*-(4-chloro-3-nitrophenyl)-*L*-cysteine as the major metabolite (Ohnishi et al., 2004). This metabolite is a product of glutathione conjugation.

#### 2,4-Dinitroanisole (DNAN)

Unlike its metabolite (2,4-dinitrophenol), DNAN has been shown to cause testicular toxicity in several animal models (Lent et al., 2018). For instance, a long-term study in rats (90 days at 80 mg/kg/day) resulted in atrophy of the seminiferous tubules. In another study in rats, after 14 days of 50 mg/kg/day, signs of testicular toxicity included decreased testicular weight (this was not a statistically significant observation, but it does corelate with other findings) and degradation of the germinal epithelium. This study identified the Sertoli cells to be most sensitive to the toxic effects of DNAN.

### 3.5 AOP: Glutathione (GSH) decrease leading to male fertility toxicity

#### 3.5.1 Evidence supporting the AOP

**Fig. 4:**
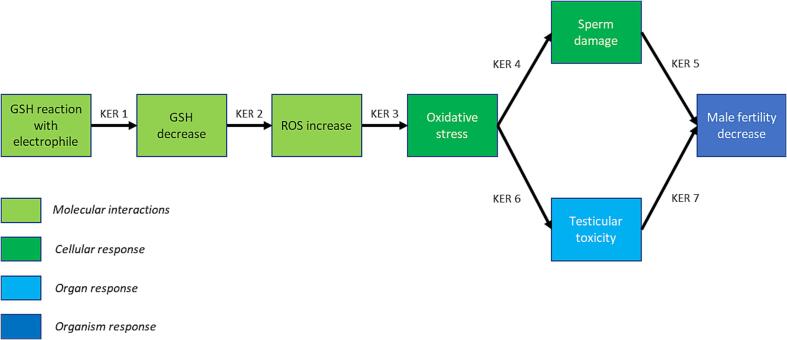
Depiction of the AOP for ‘glutathione reaction with an electrophile’ leading to ‘decreased male fertility’. GSH, glutathione; KER, key event relationship; ROS, reactive oxygen species.

The evidence used to support this pathway (Fig. 4) has primarily been generated from rodent studies examining the toxic potential of nitroaromatics. As a class, nitroaromatics are known to cause an increase in reactive oxygen species (ROS) and oxidative stress (Kovacic and Jacintho, 2001, Kovacic and Somanathan, 2014). Several compounds in this class (such as 2-*sec*-butyl-4,6-dinitrophenol, 4,6-dinitro-o-cresol, trinitrotoluene and nitrobenzene) have demonstrated testicular toxicity and impaired spermatogenesis in rodent studies (Bond et al., 1981, Levine et al., 1984, Matsumoto et al., 2008).

A range of other compounds capable of generating ROS are also associated with male reproductive toxicity (Creasy and Chapin, 2018, Kovacic and Jacintho, 2001). In addition to this, ROS generation and oxidative stress can cause sperm and testicular damage in humans, potentially resulting in reduced fertility (Asadi, 2017, Wagner et al., 2018, Wright et al., 2014).

#### 3.5.2 Summary of the role of glutathione

GSH is a tripeptide responsible for the removal of electrophilic xenobiotic compounds (through covalent binding) and quenching of ROS (Forman et al., 2009). ROS are important for a range of normal biological functions, including acting as a secondary messenger in signal transduction and mediating processes involved in ion transport, immunological host defence, transcription, and apoptosis (Ray et al., 2012, van Gelder et al., 2010). Endogenous ROS can be generated from, and play an important role in, mitochondrial oxidative phosphorylation (Ray et al., 2012). However, an excess of ROS can lead to a state of oxidative stress, which has been implicated to cause a wide range of toxic outcomes, including testicular toxicity and impairment of spermatogenesis (Creasy and Chapin, 2018, van Gelder et al., 2010). Excessive ROS and oxidative stress can be promoted by exogenous compounds, such as nitroaromatics (Creasy and Chapin, 2018, Kovacic and Jacintho, 2001). Therefore, glutathione is critical for protecting cells against excess ROS generated by xenobiotics (Forman et al., 2009). Oxidative stress is thought to play a key role in infertility in humans (Turner and Lysiak, 2008).

#### 3.5.3 Regional specificity

Oxidative stress can result in numerous types of toxicity. For instance, nitroaromatic compounds which have been proposed to cause their toxicity via oxidative stress have been implicated in causing carcinogenicity, mutagenicity, hepatotoxicity, cytotoxicity, and reproductive toxicity (Kovacic and Jacintho, 2001, Kovacic and Somanathan, 2014, Wagner et al., 2018). With respect to reproductive toxicity, oxidative stress resulting in testicular lesions has been described as a regular feature of testicular toxicants and tissue sensitivity to oxidative stress can occur (Creasy and Chapin, 2018). One hypothesis proposed for the differing tissue sensitivity is due to varied mitochondrial matrix protein expression within the mitochondria of different cell types (Creasy and Chapin, 2018). Testicular mitochondria express several signalling factors (e.g. MAPK, the Jun N-terminal kinases and p38 family members) responsible for cell death, survival, migration and division. In the testes, the above signalling factors have also been shown to regulate the junctions, which attach germ cells to Sertoli cells and the junctions which form blood-tubule barriers between Sertoli cells (Creasy and Chapin, 2018). In addition to cellular components, oxidative stress can impact on these signalling factors leading to disruption of their testicular-specific roles. In addition, sperm contain many unsaturated lipids and as a result are also sensitive to oxidative stress (Wagner et al., 2018). Finally, high rates of cell replication related to spermatogenesis are reliant on high rates of mitochondrial oxygen consumption in the germinal epithelium (Aitken and Roman, 2008) – this may make the testis more sensitive, than other organs, to oxidative stress related toxicity.

#### 3.5.4 Key event relationships

*KER 1: Glutathione reaction with an electrophile -> Glutathione decrease.*

GSH is an endogenous tripeptide compound which – through its sulfhydryl containing cystine residue – is involved in the metabolism of reactive xenobiotic compounds and quenching of ROS (Forman et al., 2009, Nepovím et al., 2004). The covalent binding of exogenous electrophilic compounds to GSH can be catalysed by the enzyme glutathione S-transferase and the resulting modified xenobiotic can be excreted from cells (Forman et al., 2009). Nitroaromatics can undergo metabolism and form functional groups that are capable of reacting with GSH (Kovacic and Somanathan, 2014). Depending on the substitution pattern of the aryl compound, nucleophilic aromatic substitution (SNAr) reactions between GSH and a nitroaromatic can also occur (Ruzza and Calderan, 2013). In addition, dimerisation of GSH can be promoted by xenobiotics capable of forming ROS, as GSH dimerisation is a consequence of enzymatic pathways involved in processing ROS (Nimse and Pal, 2015). GSH is involved in a catalytic cycle alongside glutathione oxidase, glutathione reductase and GSH-Px to cycle between GSH and the reduced form of GSH, GSSG (Forman et al., 2009, Nimse and Pal, 2015). This catalytic reduction of GSH enables the quenching of ROS.

Excessive concentrations of electrophilic xenobiotics and ROS generation can lead to depletion of glutathione. For instance:

• In the case of 1,3-dinitrobenzene, the nitro groups can be metabolised into nitroso and hydroxylamine groups and the resulting compounds can react with and deplete mitochondrial GSH (Kovacic and Somanathan, 2014).
• Experiments in mice treated with 3-methyl-4-nitrophenol also resulted in a decrease in glutathione levels (Bu et al., 2012). This decrease in glutathione was alleviated in mice treated with both 3-methyl-4-nitrophenol and the antioxidant, quercetin.
• In addition, serum and testicular GSH concentrations in rats decreased dramatically after prolonged (4 weeks) treatment with 4-nitrophenol (Zhang et al., 2015).

*KER 2: Glutathione decrease -> Reactive oxygen species increase.*

GSH is the cell’s primary defence against excessive ROS generation (Nimse and Pal, 2015). Therefore, excessive depletion of glutathione resulting from chemical insult can lead to an increase in ROS. Often prior to glutathione binding, xenobiotic species undergo transformations that can generate free radical intermediates (Wells et al., 2009). In addition to eventual conjugation with GSH, these free radical intermediates can themselves generate additional ROS which also contribute to oxidative stress. Therefore, GSH depletion through xenobiotic metabolism leads to excess ROS generation, as the cell would have reduced capacity to remove ROS. For instance:

• A reduction in glutathione observed in mice treated with 3-methyl-4-nitrophenol was also coupled with an increase in hydroxyl radicals and hydrogen peroxide (Bu et al., 2012). These increases were alleviated through co-administration with an antioxidant.
• Rats dosed with 4-nitrophenol demonstrated a significant decrease in testicular GSH, as well as a significant increase in testicular hydrogen peroxide (Zhang et al., 2015). This increase in hydrogen peroxide was alleviated by administration with an antioxidant.

*KER 3: Reactive oxygen species increase -> Oxidative stress.*

ROS are important for numerous biological processes such as mitochondrial oxidative phosphorylation, as well as mediating processes involved in ion transport, immunological host defence, transcription, and apoptosis (Ray et al., 2012, van Gelder et al., 2010, Wagner et al., 2018). Predominantly, ROS are generated in the mitochondria during oxidative phosphorylation (Ray et al., 2012). Oxidative stress is defined as an imbalance of ROS and antioxidant defences (Betteridge, 2000). Therefore, an increase in ROS to the point where the systems maintaining redox homeostasis are overwhelmed, can result in oxidative stress.

*KER 4: Oxidative stress -> Sperm damage.*

During oxidative stress, ROS covalently bind to DNA, protein and lipids, resulting in damaged cellular components and impaired cellular signalling (Creasy and Chapin, 2018, van Gelder et al., 2010). As oxidative stress can lead to toxicity *via* multiple pathways, understanding the exact mechanism of toxicity is non-trivial. Oxidative stress has been demonstrated to impair sperm function and disrupt spermatogenesis (Asadi, 2017, Wagner et al., 2018). Unsaturated fatty acids within sperm are sensitive to oxidative stress, resulting in membrane damage (Wagner et al., 2018). Radical activation of unsaturated fatty acids within the membrane of sperm can promote a chain reaction, leading to oxidation of > 50% of the plasma membrane and the generation of mutagenic species (malondialdehyde and 4-hydroxynonenal). This oxidative damage to the sperm membrane and mitochondrial DNA has been proposed to be the cause of reduced sperm motility seen in sperm exposed to ROS. Sperm are also sensitive to oxidative stress as they have limited DNA repair mechanisms. Additionally, studies have shown that humans with oligospermia correlate with higher levels of ROS (Agarwal et al., 2014, Wagner et al., 2018). Increased concentrations of oxygen (and therefore ROS) correlate with a reduction in sperm motility (Kovacic and Jacintho, 2001). Co-administration with the antioxidant enzyme CAT returned sperm motility to normal. Data also exists linking nitroaromatics, a class capable of causing oxidative stress (Kovacic and Somanathan, 2014), to sperm damage. For example:

• Mice dosed with 3-methyl-4-nitrophenol demonstrated oxidative stress in the testes which resulted in a loss of 40% of the round germ cells, and no elongated sperm were detected in a histological study (Bu et al., 2012). Co-administration with the antioxidant quercetin reversed the observed toxicity.
• Treating rats with 2,4-dinitrophenol resulted in significant reduction in spermatogonia during spermatogenesis, compared to controls (Takahashi et al., 2009).
• 2-*sec*-Butyl-4,6-dinitrophenol has been shown to cause abnormal sperm motility and morphology in rats (Matsumoto et al., 2008).
• Rats treated with trinitrotoluene had fewer spermatozoa, spermatids and spermatocytes compared to controls (Levine et al., 1984). Similar observations were seen with rats treated with nitrobenzene (Bond et al., 1981).
• Decreased spermatogenesis and testicular atrophy have been observed in rats treated with 1,3-dinitrobenzene (Cody et al., 1981).
• Decreased spermatogenesis and testicular atrophy were also observed in dogs and mice treated with 2,6-dinitrotoluene and rats treated with 2,4-dinitrotoluene (Rickert et al., 1984).

*KER 5: Sperm damage -> Male fertility decreased.*

The formation of competent sperm capable of fertilisation occurs through spermatogenesis (Schoenwolf et al., 2014). Disruption to this process could lead to a reduction of both the total number and quality of sperm produced. Adverse changes to sperm parameters can negatively impact male fertility (Wright et al., 2014). For example, high levels of DNA fragmentation are heavily associated with reduced fertility in human males. It is worth noting that male infertility can be reduced through alterations to sperm parameters alone, and do not have to be associated with other adverse events in the testes (Creasy and Chapin, 2018). Observations from rodent studies need to be considered alongside the rodent’s large reserve capacity for spermatogenesis (Creasy and Chapin, 2018). This capacity results in rodents being less sensitive for identifying male reproductive toxicants when examining sperm parameters. Rats have been shown to be capable of reproduction even with a 98% decrease in their normal sperm numbers. However, even though they remain capable of reproduction, other parameters such as litter size can be affected. Therefore, reduced fertility in rodents needs to be assessed at the population level. In humans, spermatogenesis is much more inefficient and therefore perturbation is more likely to lead to reduced fertility in comparison to rodents.

*KER 6: Oxidative stress -> Testicular toxicity.*

During oxidative stress, ROS can covalently bind to DNA, protein and lipids, resulting in damage to cellular components and impaired cellular signalling (Creasy and Chapin, 2018, van Gelder et al., 2010). As stated previously, oxidative stress can lead to toxicity *via* multiple pathways and, therefore, elucidating the exact mechanism of toxicity is non-trivial. Many compound classes of reproductive toxins are known to cause oxidative stress, which is thought to contribute to their toxicity (Creasy and Chapin, 2018, Kovacic and Jacintho, 2001). As an example, nitroaromatic compounds can cause oxidative stress (Kovacic and Somanathan, 2014) and also induce testicular toxicity. For instance:

• Mice dosed with 3-methyl-4-nitrophenol demonstrated oxidative stress in the testes, resulting in severe testicular damage including damage to the seminiferous tubules and increased testicular atrophy (Bu et al., 2012). Co-administration with the antioxidant quercetin decreased the observed toxicity. Rats treated with 3-methyl-4-nitrophenol resulted in reduced weights of the epididymis, seminal vesicle, and Cowper gland when compared to the control (Li et al., 2006). However, the weight reduction in rats was not dose dependent. Treated animals also presented decreased concentrations of testosterone, which was proposed to result from direct insult to the testes.
• 2-*sec*-Butyl-4,6-dinitrophenol has been shown to cause testicular toxicity, including decreased organ weights and atrophy in both rats and mice using gavage and feeding approaches to administer the compound (Matsumoto et al., 2008). Within the same review article, it was concluded that 4,6-dinitro-o-cresol manifested similar adverse outcomes but was less potent.
• A review article has highlighted that the testes are sensitive to nitrofurantoin, nitrofurazone, nitroimidazoles, dinitrotoluene, trinitrotoluene and chlorodinitrobenzene (Creasy and Chapin, 2018).
• Mechanistic studies have shown 1,3-dinitrobenzene to cause ultrastructural vacuolation in the Sertoli cells (Creasy and Chapin, 2018). Rats are more susceptible to 1,3-dinitrobenzene induced testicular toxicity than hamsters. This species variation has been proposed to result from a faster exhaustion of mitochondrial glutathione and adenosine triphosphate (ATP) in rats, leading to oxidative stress and testicular toxicity in rats but not in the hamster.
• An additional mechanistic study, examining the mechanism by which 1,3-dinitrobenzene can cause germinal cell damage (apoptosis) identified mitochondrial pathways as being relevant (Muguruma et al., 2005). In this study a single dose of 1,3-dinitrobenzene resulted in germinal cell apoptosis – the authors had two hypotheses for the mechanism of this: mitochondrial pathway related mechanisms and the death ligand/receptor pathway. Examining the gene expression patterns of affected testicles indicated that proteins involved in the mitochondrial pathways were irregular, whilst the death ligand/receptor relevant proteins were not affected. It could be inferred that oxidative stress in the mitochondrial may be the cause of these observations.
• Testicular toxicity has been observed in mice treated with 1,4-dichloro-2-nitrobenzene (Yamazaki et al., 2005b). In a separate study, a glutathione conjugate of 1,4-dichloro-2-nitrobenzene was identified as a major metabolite in urine of rats treated with 1,4-dichloro-2-nitrobenzene (Ohnishi et al., 2004). This indicates that testicular toxicity may have occurred as a result of glutathione depletion and therefore oxidative stress.

*KER 7: Testicular toxicity -> Male fertility decreased.*

Toxicity occurring in the testes such as atrophy is an adverse endpoint for male reproductive toxicity (Arzuaga et al., 2019, Creasy and Chapin, 2018). Adverse events occurring in the male reproductive organs have the potential to reduce male fertility.

#### 3.5.5 Essentiality

Two studies described in the section 3.4 provide evidence to support the essentiality of several key events within the above-described pathway. The study in which mice were treated with 3-methyl-4-nitrophenol demonstrated a decrease in glutathione concentrations, abnormal spermatozoa and testicular atrophy (Bu et al., 2012). Co-administration of 3-methyl-4-nitrophenol with an antioxidant alleviated the above manifestations. Similar observations were made in rats treated with 4-nitrophenol alone when compared to rats treated with 4-nitrophenol and an antioxidant (Zhang et al., 2015). These studies therefore provide evidence of the essentiality of glutathione depletion and oxidative stress, within this AOP.

#### 3.5.6 Uncertainties

ROS generation can occur through multiple pathways, including through metabolism of xenobiotics by cytochrome P450s and irradiation (Kovacic and Jacintho, 2001). GSH is a key component for quenching ROS and can support the removal of xenobiotic compounds capable of generating ROS (Forman et al., 2009). As ROS generation can occur through multiple mechanisms, the predominant mechanism leading to oxidative stress can be difficult to determine. However, this AOP focuses on glutathione depletion as a key event leading to toxicity. As glutathione is responsible for the removal of ROS, other mechanisms which lead to increased levels of ROS will also lead to depletion of glutathione and eventually oxidative stress.

Many different antioxidants can reduce ROS concentrations (Wright et al., 2014). These include enzymes such as GSH-Px, SOD and CAT, as well as small molecules such as carotenoids (Nimse and Pal, 2015). Therefore, the regulation of ROS is a process influenced by many factors.

In most cases where male fertility has been adversely affected by chemical insult, numerous specific adverse events will be observed. The profile or spectrum of adverse events can give an indication of the toxicity mechanisms that are occurring (Creasy and Chapin, 2018). The sperm damage observed in studies used to support this pathway may be a direct result of oxidative stress on the sperm, or it may occur as a result of testicular toxicity impairing the testicles’ ability to sustain healthy spermatozoa. It is also possible that the sperm damage occurs through a combination of the two. Evidence relating to 1,3-dinitrobenzene and 2,4-dinitroanisole indicates that Sertoli cell damage (testicular toxicity) causes germ cell apoptosis (sperm damage) (Ludwig et al., 2012, Takahashi and Matsui, 1993). However, similar studies were not identified for the other compounds supporting the pathway and, therefore, no direct KERs between testicular toxicity and sperm damage were included in this pathway.

2,4-DNT (and potentially other compounds) used to support this AOP are known uncouplers of oxidative phosphorylation (Terada, 1990). A process in which the proton differential across the mitochondrial inner membrane (generated by the electron transport chain) is disrupted – leading to a decrease in ATP generation (Terada, 1990). This process can also lead to an increase in ROS and oxidative stress, and could be the main driver of oxidative stress rather than direct reactivity with glutathione, or metabolism, leading to an increase in ROS.

### 3.6 Enhancing the AOP model

**Fig. 5:**
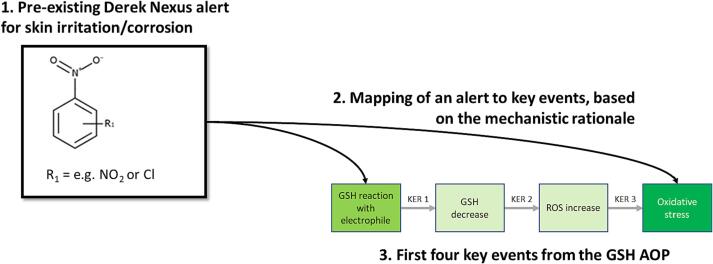
A depiction of how a pre-existing Derek Nexus alert was identified and mapped to KEs within the newly developed AOP (chemical structure information is given for the example alert). The Derek Nexus alert description references both glutathione conjugation and oxidative stress as potential mechanisms leading to toxicity. This alert captures all 13 compounds used to support the development of the GSH AOP (Table 3). AOP, adverse outcome pathway; GSH, glutathione; KER, key event relationship; ROS, reactive oxygen species.

The development of a new AOP leading from glutathione depletion to male fertility toxicity enables the supplementation of the existing AOP network with new knowledge, and provides additional KEs to which SAR alerts could be associated. The AOP model can be improved by either developing SAR alerts to predict for KEs within this AOP, or by linking existing alerts to these KEs based on mechanistic understanding. The first four KEs in the newly synthesised pathway describe a mechanism common to a wide range of adverse outcomes – for instance, oxidative stress is associated with carcinogenicity and hepatotoxicity (Klaunig and Kamendulis, 2004, McGill et al., 2014). Derek Nexus contains SAR alerts for multiple endpoints including skin sensitisation, carcinogenicity and hepatotoxicity. Some of the alerts for these endpoints may be related to chemical classes which cause their toxicity through oxidative stress related mechanisms; therefore, these could be relevant to the first four KEs in this AOP (Fig. 4). Relevant SAR alerts contained within Derek Nexus were identified by searching for terms such as “oxidative stress”, “reactive oxygen species” and “glutathione depletion” in the expert commentary. The search identified three alerts which related to nitroaromatic compounds. These alerts were initially developed for the endpoints of carcinogenicity, hepatotoxicity, and skin irritation/corrosion. The alerts all described mechanisms relevant to the first four KEs within the AOP in Fig. 4, and the alerts capture all of the compounds (Table 3) used to develop the AOP. The mechanistic information within these alerts allowed for the extrapolation and linking of the alerts to KEs within the GSH pathway described in this manuscript, introducing a predictive element to the AOP (Fig. 5). Linking alerts in such a way can increase coverage of the predictive model; however, care must be taken to ensure that the context of the alerts and key events are sufficiently related, so that relevant information is presented to safety assessors.

In addition to the three alerts described above, the key word-based review of Derek Nexus alert descriptions identified a further 73 alerts which could be mapped to the first four KEs. These alerts related to structures other than nitroaromatic compounds and, therefore, association of these alerts to the relevant KEs would enhance the predictivity of the AOP by broadening the chemical space beyond the nitroaromatic cluster used to support the generation of the pathway. Confidence in association of these alerts to the pathway would need to be reviewed on an alert-by-alert basis; however, the AOP provides an efficient means of linking and re-purposing pre-existing alerts in order to infer the potential male fertility toxicity of a compound. The GSH pathway and associated Derek Nexus alerts identified above could be integrated into the DART AOP network and DART AOP model, potentially leading to its enhanced performance.

## 4 Discussion and conclusion

A DART AOP has been developed for glutathione depletion leading to male fertility toxicity, using a dataset of TG-421 and TG-422 studies to test existing expert-based systems. This AOP has filled a knowledge gap within an existing DART AOP network. Mechanistic information within Derek Nexus alerts allowed for the association of pre-existing SAR alerts (developed for non-DART endpoints) to various KEs within the pathway, enhancing the AOP predictive model. This pathway may be useful for hazard assessment, as oxidative stress within the testicles is thought to be a common cause of male infertility (Turner and Lysiak, 2008). The evidence supporting this pathway is based on publicly available (mechanistic or fertility toxicity) studies involving nitroaromatic compounds. Despite the relatively homogenous group of chemicals used to support this pathway, it is believed that the pathway is likely relevant to a much broader range of chemicals. This assertion is, in part, supported by the structural alerts which could be linked to the pathway – these included, but were not limited to, nitroaromatic compounds. AOPs are living documents and as such could be supplemented with additional supporting evidence for non-nitroaromatic compounds. Recently, other research which aims to prove the relationships between glutathione depletion and fertility toxicity has appeared in the public domain (Vieira et al., 2023, Zhang, 2023). These AOP documents are not yet complete, and appear to relate to evidence from non-nitroaromatic compounds (such as atrazine). Once completed, the evidence within these documents could enhance the overall weight of evidence linking glutathione depletion to testicular toxicity – this could potentially support one overall pathway.

The dataset of TG-421 and TG-422 studies (Yamada et al., 2021) provided insight into the performance of the AOP model. The availability of such data is important as it provides a valuable means for researchers to develop and enhance knowledge bases. This validation highlighted an improved performance (in terms of sensitivity) when comparing the AOP model to the Derek Nexus DART model. This improved performance is likely due to the broadening of the endpoints covered in the DART AOP model compared to Derek Nexus alone, and also the ability that the AOP-based predictive model provided to incorporate a broader suite of SAR alerts. The curated dataset also proved useful in the identification of predictive/knowledge gaps within a developing AOP network. Investigation of these gaps provided valuable insight into mechanisms of toxicity not yet captured by the AOP network. These gaps were identified through analysis of clusters of false negative compounds – several of which were identified as useful to focus on to further improve the knowledge within the AOP network. Further investigation into one of these clusters of chemicals led to the development of an AOP documenting a mechanism of glutathione depletion leading to reduced male fertility (Fig. 4). There are a number of data mining approaches that can be used to probe the coverage of existing knowledge. This study demonstrated the value of expert knowledge when it comes to extracting meaningful signals from cheminformatic and bioinformatic approaches – ensuring that such signals are translated into meaningful assets (e.g. AOPs) that can be used to enhance knowledge.

Literature review of the cluster of compounds described in Table 2 focused on identifying additional DART-relevant data and mechanistic information which could rationalise the DART findings. The literature review focused on nitroaromatic compounds as these comprised the majority of compounds in Table 2, and the search was broadened to include studies involving other structurally similar nitroaromatic compounds. Many of these substances induce sperm damage and testicular toxicity. Furthermore, some were observed to deplete GSH and increase ROS. These pathways did not exist in the AOP network; therefore, it was possible to make an AOP based on the cluster initially identified. These compounds often resulted in a broad range of toxicity – this is likely due to the mechanism of toxicity involving oxidative stress (which can affect many organs) (Deavall et al., 2012). This may explain why most of the toxicants in Table 2 were non-specific and why several of the non-toxicants (according to TG-421 and TG-422) had a relatively low highest-tested dose (suggested upper dose limit for the studies was 1000 mg/kg (OECD, 2016, OECD, 2015)). It may also explain why limited data were available to provide support for the development of a female fertility- or embryo toxicity-based AOP (based on nitroaromatic compounds). Additionally, a review of the sensitivity of spermatozoa and oocytes to oxidative stress indicates that oocytes are sensitive; however, high or long-term exposure may be needed to observe relevant toxicity (Aitken, 2020). This may not have been possible with the generally toxic nitroaromatic compounds in traditional animal studies. Also, in the case of embryo toxicity, it may be difficult to distinguish whether the toxicity is a result of a primary or secondary (maternal) toxicity.

Based on the supporting evidence, the AOP (Fig. 4) is likely applicable to multiple species, including humans. While oxidative stress is a ubiquitous process in all cells, differential gene expression in testicular tissue is possibly responsible for the sensitivity of this organ to this adverse effect, thus accounting for regional specificity (Creasy and Chapin, 2018). Although oxidative stress is shown to be associated with both cellular sperm damage and testicular organ toxicity, both plausibly impact male fertility, hence why both are included in one AOP. The AOP provided a mechanistic rationale which allowed for the mapping of multiple pre-existing Derek Nexus alerts (for various non-DART endpoints) to relevant KEs. Three of these Derek Nexus alerts capture the nitroaromatic compounds used to develop the AOP, as their alert descriptions indicated that these compounds can bind to GSH, causing ROS and oxidative stress. Validation of these alerts against the donated dataset was not performed due to the differing endpoints between the AOP and the donated data (male fertility toxicity vs female fertility and developmental toxicity). However, the performance of these alerts could be validated against a dataset for testicular toxicity, glutathione reactivity, or oxidative stress. Once mature, this network may be a suitable resource to help incorporate QSAR models based on non-animal data into a toxicity screen (e.g. (Myden et al., 2022)), and to contextualise results from alternative assays and drive the development of IATAs. This could potentially improve the quality of decision-making during DART-based safety assessments when used in combination with other data, evidence and knowledge, thus reducing the burden to run *in vivo* studies. The collaboration between the NIHS of Japan and Lhasa Limited allowed for the testing and enhancement of DART AOP knowledge. The chemical space contained within the dataset related to high-volume chemicals, whereas the AOP network had predominantly been built on the pharmaceutical chemical space. This therefore provided a valuable and novel resource which allowed for the broadening of AOP knowledge – achieved through the development of the AOP within this manuscript. This work also demonstrates how collaborations are valuable for driving scientific progress.

## Funding

This study was supported by the National Institute of Health Sciences (NIHS) of Japan, which was funded by a Health and Labour Science Research Grant 21KD2005. The funding provider (Ministry of Health, Labour and Welfare) had no further involvement in the work undertaken within this manuscript.

## CRediT authorship contribution statement

**Alun Myden:** Investigation, Writing – review & editing, Writing – original draft. **Susanne A. Stalford:** Conceptualization, Writing – review & editing, Writing – original draft. **Adrian Fowkes:** Conceptualization, Investigation, Writing – review & editing, Formal analysis. **Emma White:** Data curation, Investigation, Writing – review & editing. **Akihiko Hirose:** Conceptualization, Writing – review & editing. **Takashi Yamada:** Conceptualization, Writing – review & editing, Funding acquisition.

## Declaration of Competing Interest

The authors declare that they have no known competing financial interests or personal relationships that could have appeared to influence the work reported in this paper.

## Data Availability

The data that has been used is confidential.
